# Improved furfural tolerance in *Escherichia coli* mediated by heterologous NADH-dependent benzyl alcohol dehydrogenases

**DOI:** 10.1042/BCJ20210811

**Published:** 2022-05-23

**Authors:** Benjamin James Willson, Reyme Herman, Swen Langer, Gavin Hugh Thomas

**Affiliations:** 1 Department of Biology, University of York, York YO10 5DD, U.K.; 2 Technology Facility, Department of Biology, University of York, York YO10 5DD, U.K.

**Keywords:** *Acinetobacter calcoaceticus*, benzyl alcohol dehydrogenase, *Burkholderia ambifaria*, furfural tolerance, lignocellulosic feedstocks, *Pseudomonas putida*

## Abstract

While lignocellulose is a promising source of renewable sugars for microbial fermentations, the presence of inhibitory compounds in typical lignocellulosic feedstocks, such as furfural, has hindered their utilisation. In *Escherichia coli*, a major route of furfural toxicity is the depletion of NADPH pools due to its use as a substrate by the YqhD enzyme that reduces furfural to its less toxic alcohol form. Here, we examine the potential of exploiting benzyl alcohol dehydrogenases as an alternative means to provide this same catalytic function but using the more abundant reductant NADH, as a strategy to increase the capacity for furfural removal. We determine the biochemical properties of three of these enzymes, from *Pseudomonas putida*, *Acinetobacter calcoaceticus*, and *Burkholderia ambifaria*, which all demonstrate furfural reductase activity. Furthermore, we show that the *P. putida* and *B. ambifaria* enzymes are able to provide substantial increases in furfural tolerance *in vivo,* by allowing more rapid conversion to furfuryl alcohol and resumption of growth. The study demonstrates that methods to seek alternative cofactor dependent enzymes can improve the intrinsic robustness of microbial chassis to feedstock inhibitors.

## Introduction

In recent years, lignocellulose has become increasingly apparent as a potential renewable feedstock for bioprocessing [1]. Lignocellulose is a complex material, found in plant cell walls, which consists primarily of three major classes of polymer: cellulose, a polymer of glucose joined by β-(1,4) glycosidic bonds, which forms a crystalline structure; hemicellulose, a heterogeneous group of polysaccharides, typically polymers of d-xylose, d-glucose or l-arabinose with various degrees of substitution; and lignin, a polymer of phenolic compounds [2]. Enzymatic hydrolysis of cellulose and hemicellulose yields sugars, which can be used as a feedstock for microbial growth [1]. However, lignocellulosic biomass is extremely recalcitrant to enzymatic saccharification [3], and must be pre-treated to remove lignin and hemicellulose and to disrupt the cellulose crystal structure [4]. This pre-treatment typically leads to the formation of toxic compounds. One of the more significant of these is furfural, an aromatic, heterocyclic aldehyde which is formed from pentose sugars such as D-xylose when heated in the presence of acids [5]. Thus, furfural is produced from the pentose fraction of hemicellulose when subjected to pre-treatments such as dilute acid [6] or steam explosion [7]; while more severe pre-treatments solubilise greater amounts of hemicellulose, they also result in the production of greater levels of furfural.

A number of routes have been implicated in the mechanism of furfural toxicity [8]. In *Saccharomyces cerevisiae* [9] and *Escherichia coli* [10], furfural has been associated with the accumulation of reactive oxygen species. Furfural is also capable of directly damaging DNA *in vitro* [11], and has been identified to react with DNA at AT sites [12]. This observation could be explained by a proposed reaction between furfural and adenine, forming kinetin [13], which has been shown to promote the mismatching of bases during polymerisation when incorporated into DNA [14]. Some engineering strategies, such as the overexpression of *thyA* [15] or of polyamine transporters [16], have been proposed to alleviate furfural toxicity by improving DNA repair or by protecting DNA from damage respectively.

**Figure 1.**
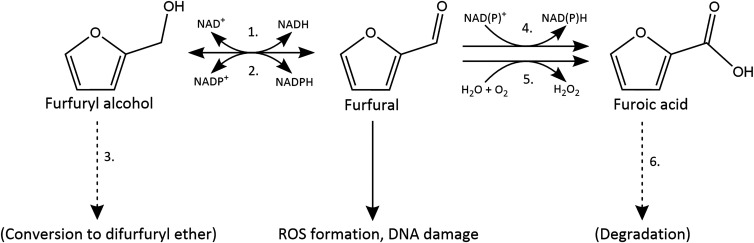
Strategies used by microorganisms for the detoxification of furfural. Furfural is converted to furfuryl alcohol by the action of NADH-dependent (1., e.g. FucO in *E. coli* [28]) or NADPH-dependent (2., e.g. YqhD in *E. coli* [22]) reductases. In *Acinetobacter baylyi* (3.)*,* furfuryl alcohol has been observed to be subsequently converted to difurfuryl ether [49]. Alternatively, furfural is oxidised to furoic acid by the action of aldehyde dehydrogenases (4., e.g. ALD6 in *S. cerevisiae* [60]) or by furfural oxidase (5., e.g. HmfH in *Cupriavidus basiliensis* [20]). In some organisms (6.), conversion to furoic acid is the first step in a degradation pathway that allows the utilisation of furfural as a carbon source [61].

In microorganisms, furfural is typically detoxified by conversion into less harmful compounds such as furfuryl alcohol and/or furoic acid (Figure 1) [17–20]. Exposure of different microbes to furfural results in decreases in intracellular NADH and NADPH levels as these are used as substrates for furfural reduction [17,21]. In *E. coli*, this is a significant cause of furfural-induced growth inhibition; furfural is converted to furfuryl alcohol primarily through the actions of NADPH-dependent reductases with a high affinity for NADPH, such as YqhD and DkgA [22], which can outcompete biosynthetic enzymes for reduced cofactor at low concentrations. One identified effect of this is the inhibition of sulphur uptake, as the assimilation of sulphate into organic sulphur requires 4 units of NADPH [23]. Correspondingly, for *E. coli*, many of the successful strategies for engineering of furfural resistance have focused on addressing the depletion of NADPH. These strategies include the identification and deletion of the furfural-active reductases that use NADPH [22], the expression of a transhydrogenase capable of replenishing the pool of NADPH [23], the rerouting of carbon flux through the pentose phosphate pathway [24,25], and the expression of reductases that are able to reduce furfural without using NADPH [26,27].

One reductase that has been shown to improve furfural tolerance in *E. coli* is the NADH-dependent lactaldehyde reductase FucO [28]. FucO expression in an ethanologenic *E. coli* strain was shown to result in a strong increase in NADH-linked furfural reductase activity, resulting in an increased MIC for furfural, increased rate of furfural detoxification, and increased growth rate when the NADPH-utilising reductase *yqhD* was also deleted. FucO was further optimised for furfural detoxification by mutagenesis, leading to the generation of a variant with increased expression and reduced *K*_M_ for furfural; this variant was shown to further improve the detoxification of furfural and the growth phenotype [29]. However, a significant potential issue with the use of FucO for furfural tolerance is that it is inactivated under aerobic conditions [30], and while the benefit of FucO has been demonstrated for non-aerated cultures, this oxygen sensitivity is a major issue for most fully aerobic bioconversion strategies. Thus, in this work, we aimed to identify a stable alternative reductase that would allow improved resistance to furfural under aerobic conditions. After examining the literature for promising candidates, we have identified NADH-linked furfural reductase activity in three enzymes from the benzyl alcohol dehydrogenase (BAD) family: *Pseudomonas putida* XylB, *Acinetobacter calcoaceticus* BAD, and *Burkholderia ambifaria* BAD. We demonstrate a substantial improvement in furfural tolerance for strains expressing XylB and *B. ambifaria* BAD, and confirm that the XylB-expressing strain showed an improved ability to reduce furfural *in vivo*, demonstrating the potential of this group of enzymes for engineering of furfural tolerance.

## Results

### Identification of novel furfural reductase candidates

To identify suitable reductase candidates, we examined the literature, including the BRENDA database [31], for enzymes with either known NADH-linked furfural reductase activity or alternatively enzymes known to catalyse NAD^+^-linked furfuryl alcohol dehydrogenase activity which can also function in the reverse reaction, for example the alcohol dehydrogenase ADH1 from *S. cerevisiae* [32]. However, most had features that might prevent their use in *E. coli*. For example, the FurX protein from *Cupriavidus necator* is able to reduce furfural efficiently, but requires ethanol as the source of reducing power [33], whereas the xylose reductase ZMO0976 from *Zymomonas mobilis* can use NADH but has a higher affinity for NADPH [34].

We identified the XylB protein from *P. putida* as a potential candidate enzyme [35]. XylB is an NAD^+^/NADH-dependent aryl alcohol dehydrogenase which belongs to the zinc-dependent alcohol dehydrogenase superfamily. While there are other enzymes from this superfamily with furfural reductase activity, such as *E. coli* YahK and YjgB [36] and *C. necator* FurX [33], XylB is to our knowledge the only enzyme from this superfamily shown to reduce furfural using NADH as a source of reducing power, making it an especially interesting target for analysis. In *P. putida*, XylB forms part of the degradation pathway for methylated aromatic compounds such as toluene and xylene. In this pathway, the methyl group is oxidised to an alcohol group, which is then converted to an aldehyde by XylB; the resulting aldehyde is further oxidised to an acid and degraded by other enzymes in the pathway. While XylB was found to be mostly active towards benzyl alcohol and substituted derivatives, furfuryl alcohol was converted to furfural at 21% of the rate of the benzyl alcohol reaction. XylB was also shown to reduce benzaldehyde to benzyl alcohol, but the ability to use furfural as a substrate for conversion to furfuryl alcohol was not tested [35]. Importantly, XylB was also identified as being particularly stable, with no effect on activity after aerobic incubation at 37°C for 21 h. We chose to include the related benzyl alcohol dehydrogenase (BAD) enzyme from *A. calcoaceticus* (AcBAD) [37] in our analysis as it is also able to oxidise furfuryl alcohol [38] and the structure of the protein has been solved (PDB: 1F8F [39]). We also included a third BAD enzyme from *B. ambifaria* (BaBAD), which has known structure (PDB: 5TNX [40]) and, although biochemically uncharacterised, is phylogenetically equidistant to XylB and AcBAD [41]. To enable biochemical characterisation, each target gene was synthesised after codon optimisation (see Methods and Supplementary Data S1) and cloned into the pBADcLIC expression vector [42].

### Purification and cofactor specificity of candidate furfural reductases

**Figure 2.**
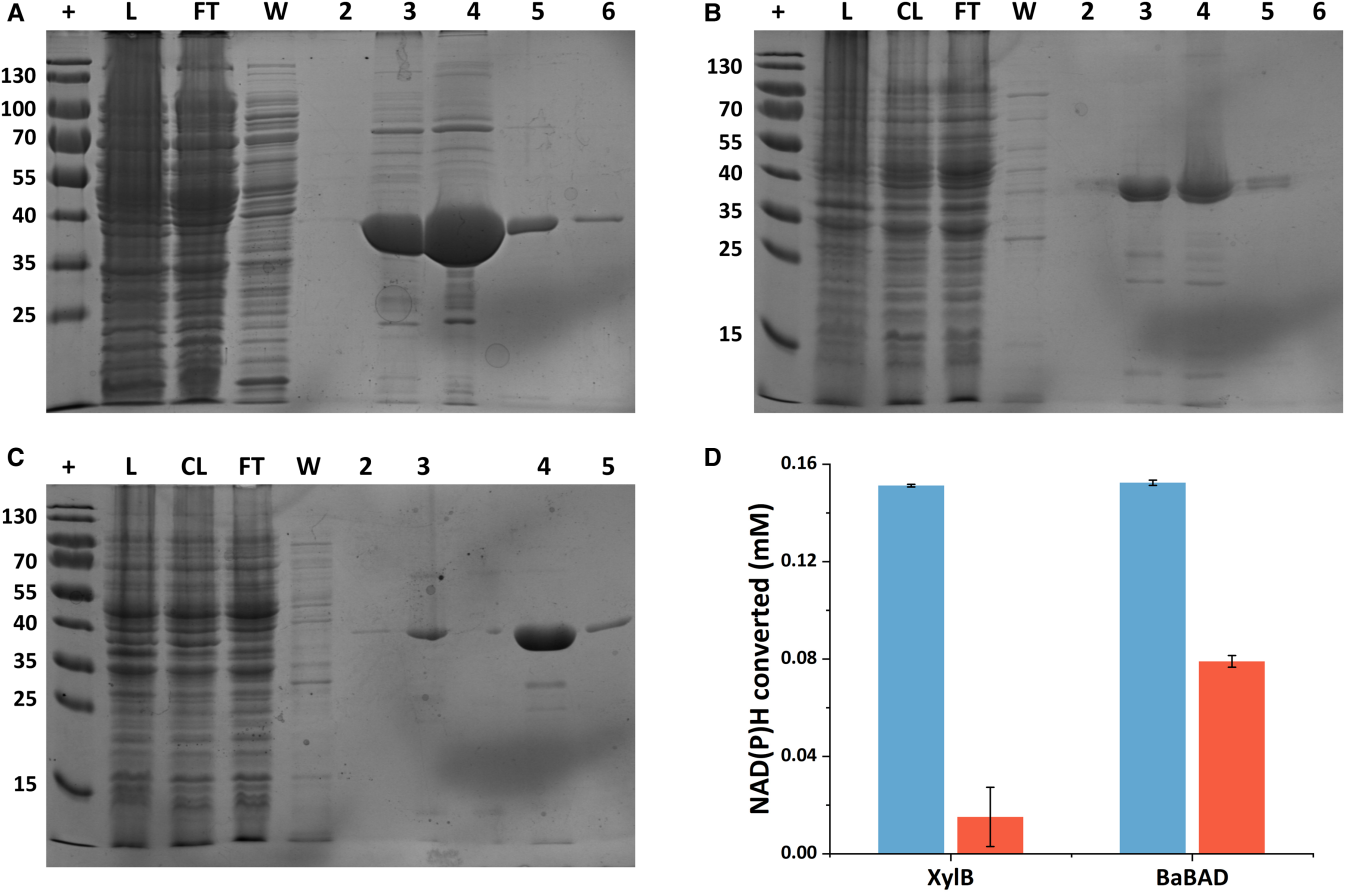
Purification of the candidate furfural dehydrogenases. (**A**–**C**) are Coomassie-stained SDS–PAGE gels containing protein separated from different stages of the purification of (**A**) *P. putida* XylB, (**B**) BaBAD and (**C**) AcBAD. The lanes are, +: ladder (PageRuler™ Prestained Protein Ladder, 10 to 180 kDa, ThermoFisher); L: lysate; CL: centrifuged lysate; FT: nickel column flow through; W: nickel column wash; 2–6: nickel column elution fractions. Sizes of ladder markers are given as molecular mass in kilodaltons. (**D**) shows activity of XylB and BaBAD with either NADH (blue) or NADPH (orange) as the electron donor in the reduction in furfural to furfuryl alcohol, measured by a change in A_340_ after 1 h.

Expression of the recombinant enzymes was assessed in *E. coli* using the pBADcLIC system, which adds a C-terminal deca-histidine tag to the protein to enable purification by nickel affinity chromatography. All three enzymes were readily purified via HPLC using a nickel affinity purification method (Figure 2). The expression levels of XylB and BaBAD appeared to be somewhat higher than that of AcBAD, although a sufficient quantity of AcBAD was purified for the assay.

Experimentally characterised examples of benzyl alcohol dehydrogenases can use NADH [43] or NADPH [44] as reductants when operating in the reverse direction. When functioning as a dehydrogenase, the *P. putida* MT-2 XylB protein is highly specific for NAD^+^ over NADP^+^ [35], and the known structure of AcBAD contains bound NADH; however, the preferred cofactor of BaBAD is not known. To examine the cofactor preference of our selected enzymes, we used a spectrophotometric assay utilising the ability of NADH and NADPH to absorb at 340 nm [45], which is reduced as the cofactor is oxidised during the conversion of the substrate to its alcohol form.

After 1 h, both XylB and BaBAD had been able to consume NADH and NADPH in the presence of furfural, demonstrating their ability to reduce furfural and that the reduction was less efficient in the presence of NADPH than with NADH (Figure 2D). After 1 h in the presence of 0.22 mM NADH and 2 mM furfural, we calculated that XylB and BaBAD had converted ∼0.15 mM NADH, which is likely to represent the end-point of the reaction. Conversely, XylB was only able to reduce the NADPH concentration by 0.015 mM in the same time, whereas BaBAD was able to reduce it by 0.079 mM. Thus, neither enzyme was able to effectively use NADPH to reduce furfural, although BaBAD did show some activity with this cofactor, unlike XylB.

**Figure 3.**
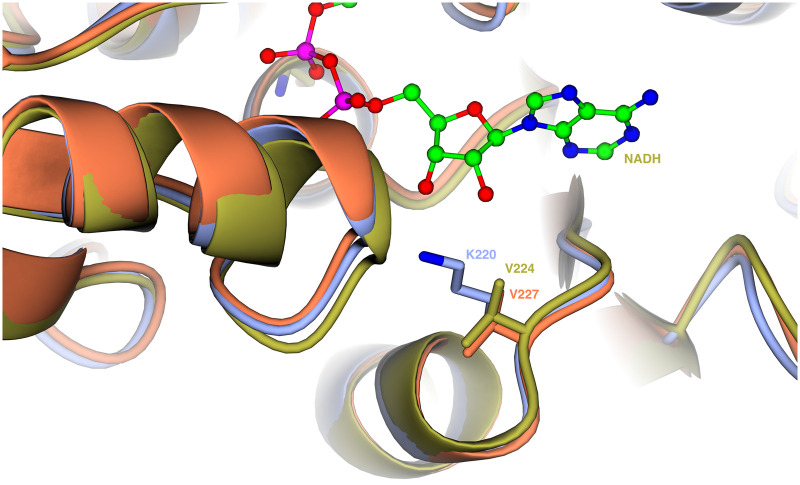
Structures of the benzyl alcohol dehydrogenase NADH binding pocket. Crystal structures of AcBAD (gold) and BaBAD (orange) are overlaid with the SWISS-MODEL prediction of XylB (blue, provided separately as Supplementary Data S3). The indicated residue (XylB_K220_/AcBAD_V224_/BaBAD_V227_) may explain the discrepancy in cofactor utilisation between XylB and BaBAD.

To understand this difference in selectivity between the enzymes, we examined the known and predicted structures, which suggest differences in the cofactor binding pocket. V227 in BaBAD is replaced with a lysine in XylB (Supplementary Data S2); in an SWISS-MODEL [46] prediction of the XylB structure (Supplementary Data S3), this residue projects into the binding pocket in the region where the phospho group of NADPH would be located, potentially restricting the binding of NADPH relative to NADH (Figure 3).

### Kinetic analysis of substrate specificity

**Figure 4.**
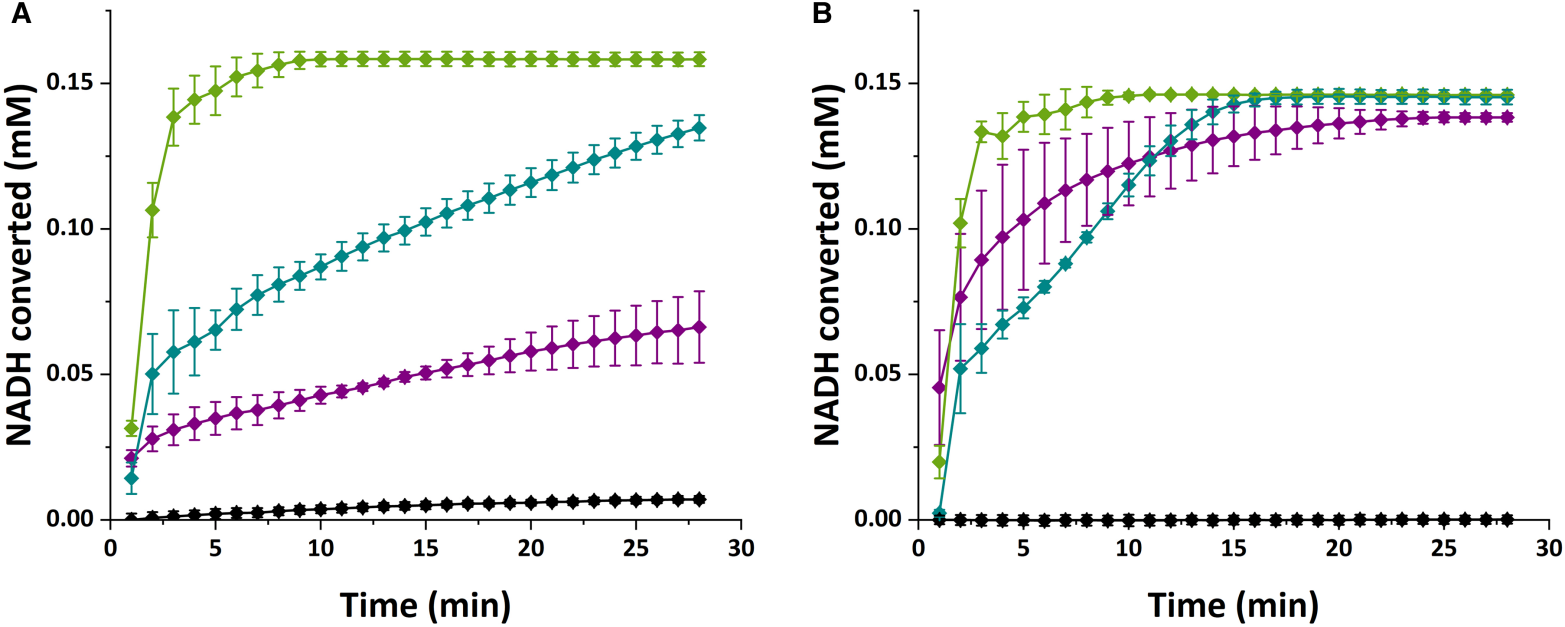
Example data from the kinetics assay. Selected data shows conversion of 0.22 mM NADH by XylB (purple), AcBAD (green), BaBAD (turquoise) or no enzyme (black) in the presence of 2 mM benzaldehyde (**A**) or 2 mM furfural (**B**). Data shows the mean of three technical replicates with error bars showing standard deviation.

Using NADH as the reductant, the three enzymes were then analysed in more detail for their activity against furfural and the related furanic compound hydroxymethylfurfural, as well as benzaldehyde as a positive control, and the aliphatic compound hexanal as a negative control. As expected, all three enzymes are active with benzaldehyde as a substrate (Figure 4, Table 1 and Supplementary Data S4). Conversely, only BaBAD was able to utilise hexanal, albeit with a low catalytic efficiency (*k*_cat_/*K*_M_). For the test compounds, furfural and HMF, we observed detectable activity for all three enzymes (Table 1), with furfural being reduced with greater catalytic efficiency than HMF. Interestingly, all three enzymes had comparable efficiencies for furfural and benzaldehyde. However, the *k*_cat_ and *K*_M_ values varied greatly. XylB showed much lower *k*_cat_ and *K*_M_ values towards benzaldehyde than the other enzymes, which may suggest that it functions more efficiently during alcohol oxidation than during aldehyde reduction. Conversely, AcBAD showed much greater *k*_cat_ and *K*_M_ values towards benzaldehyde than the other enzymes; it may be the case that the active site binds benzaldehyde more weakly, allowing faster turnover but requiring a higher substrate concentration to saturate the active site. Finally, BaBAD showed comparable *k*_cat_ and *K*_M_ values for furfural and benzaldehyde, suggesting that it interacts with both substrates in a similar manner. Perhaps the most promising from this *in vitro* data was the XylB protein, which demonstrated a higher rate constant against furfural compared with the other three substrates.

**Table 1:** Activity data of benzyl alcohol dehydrogenases for the reduction in aldehydes in the presence of NADH, showing 95% CI

	*k*_cat_ (m^−1^)	*K*_M_ (mM)	*k*_cat_/*K*_M_ (m^−1^ mM^−1^)
XylB: furfural	147 ± 28.0	0.234 ± 0.153	600 ± 400
XylB: benzaldehyde	11.5 ± 2.06	0.0212 ± 0.0162	500 ± 400
XylB: HMF	69.5 ± 2.72	0.461 ± 0.0521	200 ± 20
XylB: hexanal	ND	ND	ND
AcBAD: furfural	164 ± 28.0	0.359 ± 0.224	500 ± 200
AcBAD: benzaldehyde	394 ± 80.5	0.412 ± 0.251	1000 ± 600
AcBAD: HMF	33.9 ± 8.75	0.294 ± 0.277	100 ± 100
AcBAD: hexanal	ND	ND	ND
BaBAD: furfural	34.5 ± 6.71	0.0752 ± 0.0622	500 ± 400
BaBAD: benzaldehyde	51.9 ± 4.71	0.0783 ± 0.0300	700 ± 300
BaBAD: HMF	65.3 ± 11.5	0.377 ± 0.242	200 ± 100
BaBAD: hexanal	45.5 ± 9.77	0.672 ± 0.374	70 ± 40

ND, not determinable.

### Effect of furfural reductase expression on growth in furfural-containing medium

**Figure 5.**
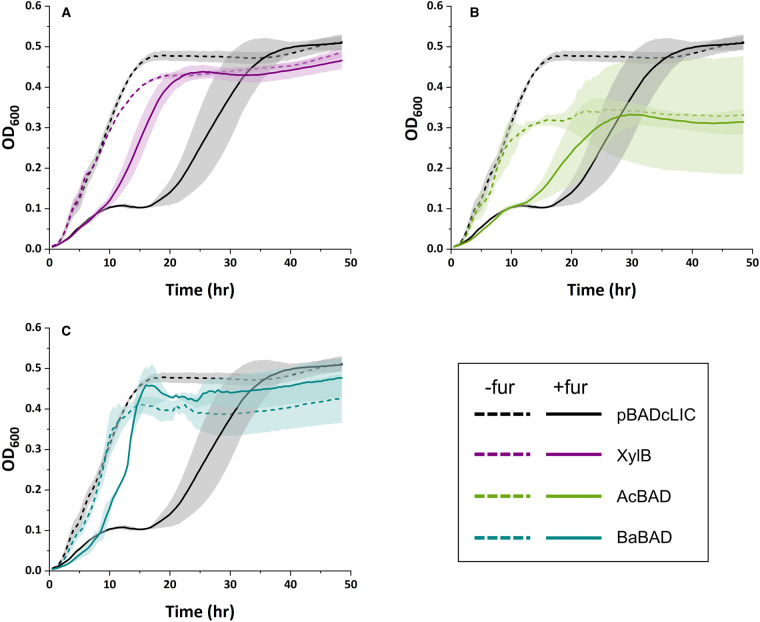
Growth of BAD-expressing *E. coli* in the presence and absence of furfural. Data shows the growth of *E. coli* BW25113 pBADcLIC (black) against: (**A**) *E. coli* BW25113 pBADcLIC_XylBfix (purple), (**B**) pBADcLIC_AcBAD (green) or (**C**) pBADcLIC_BaBAD (turquoise) in modified M9 medium (dashed line) or modified M9 medium with 10 mM furfural (solid line). OD_600_ was measured every 30 min. Line represents the mean of three biological replicates and shaded area represents standard deviation.

The results of the *in vitro* assay suggested that all three of the chosen enzymes might have the potential to confer furfural tolerance *in vivo*. Thus, the growth of all three expression strains was tested in modified M9-xylose medium containing 10 mM furfural (Figure 5). In this medium, the growth of the control strain is inhibited up until ∼18 h, at which point the furfural has presumably all been converted to furfuryl alcohol; this pattern is typically observed in *E. coli* when exposed to furfural where growth is completely inhibited until the toxin is removed, and hence the rate at which it is removed is a good indicator of activity [24]. The strains synthesising XylB and BaBAD showed substantial improvements in growth in the presence of furfural, entering exponential growth at ∼9 h, and reaching maximal OD_600_ at ∼22 h for XylB or 16 h for BaBAD. Conversely, the AcBAD-expressing strain showed a smaller increase in furfural tolerance, entering exponential phase at 12 h and showing much slower outgrowth. However, expression of AcBAD appears to be detrimental for growth, with a reduced maximal OD_600_ even in the absence of furfural. Nevertheless, the data suggests that the expression of heterologous NADH-dependent BAD enzymes is a promising route to confer furfural tolerance to *E. coli*.

**Figure 6.**
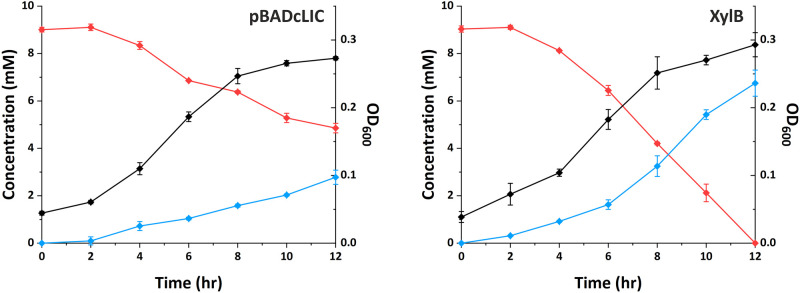
Conversion of furfural to furfuryl alcohol by *E. coli* BW25113 pBADcLIC (left) and pBADcLIC_XylBfix (right). Concentration of furfural is shown in red, furfuryl alcohol in blue, and OD_600_ in black. Furoic acid was not detected in either culture (data not shown). Data represents the mean of three biological replicates, with the exception of furfural and furfuryl alcohol in the pBADcLIC culture at 4 and 6 h, which are the mean of two replicates. Error bars show standard deviation.

To further investigate the mechanism of resistance, we undertook analysis on supernatants from the furfural removal stage of cultures grown in the presence of 10 mM furfural and followed both furfural removal and accumulation of furfuryl alcohol (Figure 6). In this assay, the strain expressing XylB was able to reduce furfural more rapidly, with furfural no longer detectable at 12 h. At this time point, ∼5 mM furfural remained in the control cultures, confirming that heterologously expressed XylB is able to increase the rate of furfural reduction *in vivo*. Interestingly, the conversion rates of both cultures is very similar for the first 6 h. However, between 6 and 12 h, the rate of conversion in the XylB-expressing culture proceeds at a faster rate than in the control, with a rate of 0.355 mM/hr (*R*^2^ = 0.9689) for the control culture and 1.07 mM/hr (*R*^2 ^= 0.9998) for the XylB-expressing culture (Supplementary Figure S1). Thus, after 6 h, the XylB-expressing strain was able to reduce furfural at approximately three times the rate of the control strain. Curiously, the 6 h time point coincides with a decrease in growth rate in both strains, which is consistent with the biphasic growth curves observed in Figure 4. One potential explanation for these observations is that furfural reduction could be dominated by NADPH-utilising reductases in the beginning of the culture, but after 6 h, NADPH production becomes insufficient to sustain the initial rates of outgrowth and furfural conversion. This would lead to a drop in the rates of growth and NADPH-linked furfural reduction in both cultures, but the XylB-expressing cultures would continue to reduce furfural at a higher rate using NADH. Furfuryl alcohol is likely to be the only product; furoic acid was not detected in any of the cultures (data not shown), which is consistent with other reports of *E. coli* [26]. Furthermore, the concentration of furfuryl alcohol in the XylB culture remained roughly constant from 12 to 24 h (Supplementary Data S5), confirming that furfuryl alcohol was not converted further by the cells. However, the amount of furfuryl alcohol detected was less than the amount of furfural removed. This could be explained by evaporation of some of the furfural; alternatively, it is possible that some of the furfural is being retained by the biomass, either through partition into the cell membrane, or by formation of adducts with DNA or with cysteine residues as has been observed *in vitro* for furfural and hydroxymethylfurfural respectively [13,47]. A final sample taken at 24 h (Supplementary Data S5) confirmed that furfural was also undetectable in the control cultures at this time and that both strains were able to grow to a similar OD_600_ (mean OD_600_: control, 4.41; XylB, 5.25).

## Discussion

In this work we aimed to find alternative improved enzymes that would catalyse the aerobic reduction in furfural to furfuryl alcohol and that would function readily in microbial cells. Our choice of enzymes identified primarily for catalysing the reverse reactions was vindicated in detection of the desired reaction both *in vitro* and *in vivo*. Although the canonical role of XylB is the formation of benzaldehyde from benzyl alcohol, it has been shown that when the Xyl pathway is expressed in *E. coli*, XylMA is able to catalyse both the oxidation of toluene to benzyl alcohol and the subsequent oxidation of benzyl alcohol to benzaldehyde, whereas coexpression of XylB results in back-formation of benzyl alcohol. Thus, under physiological conditions in *E. coli*, the back reaction is heavily favoured [48]. It was suggested that the role of XylB may be to prevent the accumulation of benzaldehyde or to allow oxidation when the concentration of benzaldehyde is very low. This may not be optimal for toluene degradation in engineered strains, but it is ideal for our purpose of furfural reduction. Furthermore, there is some evidence that these enzymes may be involved in furfural tolerance in nature. For example, in *Acinetobacter baylyi* [49], the gene *areB* was recently shown to be up-regulated by over 20-fold after the addition of furfural; the corresponding protein is a close homologue of the *A. calcoaceticus* protein used in this work, sharing 82.75% identity. *A. baylyi* was shown to convert furfural into difurfuryl ether via furfuryl alcohol, and AreB was suggested as a candidate for catalysis of the initial reduction of furfural.

All three reductases were shown to give improvements in growth in the presence of furfural. However, the final OD_600_ of the AcBAD-expressing culture was markedly lower even under control conditions, and AcBAD was less well expressed than the other two proteins, which suggests that expression of AcBAD may be toxic to the cells.

While XylB and AcBAD have been previously shown to interact with furfuryl alcohol, BaBAD has not been previously characterised to our knowledge, and was selected purely on the basis of homology and the existence of a solved crystal structure. Examination of the gene neighbourhood of BaBAD shows that BaBAD is in an operon with benzaldehyde dehydrogenase; immediately upstream in the opposite direction is a gene cluster encoding genes homologous to *benABCD* and *catBCA*, which are involved in the conversion of benzoate to catechol and subsequent conversion of catechol to 3-oxoadipate-enol-lactone [50]. Thus, the role of BaBAD appears to be the degradation of benzyl alcohol and related compounds via the catechol ortho-cleavage pathway [51].

The expression of NADH-dependent furfural reductases is one route towards the improvement of bioproduction using lignocellulosic feedstocks. Most research has focused on the use of FucO, an iron-dependent alcohol dehydrogenase, for this purpose [28,29]. Additionally, the short-chain dehydrogenase enzymes UcpA [26] and, very recently, YghA have been shown to confer improved tolerance to furfural in *E. coli* [27]. However, this work is, to our knowledge, the first demonstration of improved furfural tolerance through heterologous expression of benzyl alcohol dehydrogenases. It is probable that the family of benzyl alcohol dehydrogenases will contain uncharacterised enzymes with even greater furfural reductase activity. This work is certainly not an exhaustive screening of benzyl alcohol dehydrogenases and examining a wider variety of genes could be an interesting route for future work to take. It may also be possible to further improve the activity or specificity of these enzymes for furfural; this could be accomplished by rational engineering based on the crystal structures, directed evolution based on tolerance to furfural as a method of selection, or the saturation mutagenesis approach applied to FucO by Zheng et al. [29]. Nevertheless, the benzyl alcohol dehydrogenases can be added to the library of known protective enzymes, providing more tools for the improvement of lignocellulosic bioproduction strategies.

## Methods

### Strains, plasmids and culture conditions

Lysogeny broth (LB; 10 g/L tryptone, 5 g/L yeast extract, 10 g/L sodium chloride, pH ∼7.0) was used for routine culture of *E. coli*. For growth on solid medium, LB with 15 g/L bacteriological agar was used. Strains were stored at −80°C in LB with 20% (v/v) glycerol. Where relevant, ampicillin was added to a concentration of 100 µg/ml. Cultures were routinely incubated at 37°C and liquid cultures were shaken at 220 rpm. For a full list of strains and plasmids used in this work, see Table 2.

**Table 2:** Strains and plasmids used in this work

Plasmid	Notes	Reference
pBADcLIC	Empty expression vector. Amp^R^.	[42]
pUC57_noBsaI_XylBco	Cloning vector with codon-optimised XylB. Amp^R^.	This work; ordered from Biomatik
pBADcLIC_XylBco	Expression vector with codon-optimised XylB. Amp^R^.	This work
pBADcLIC_XylBfix	Expression vector with codon-optimised and sequence-corrected XylB. Amp^R^.	This work
pUC57_AcBAD	Cloning vector with codon-optimised AcBAD. Amp^R^.	This work; ordered from Biomatik
pBADcLIC_AcBAD	Expression vector with codon-optimised AcBAD. Amp^R^.	This work
pUC57_BaBAD	Cloning vector with codon-optimised BaBAD. Amp^R^.	This work; ordered from Biomatik
pBADcLIC_BaBAD	Expression vector with codon-optimised BaBAD. Amp^R^.	This work
Strain		
*Escherichia coli* BW25113	Δ(*araB–D*)567 Δ(*rhaD–B*)568 Δ*lacZ*4787(::*rrnB*-3) *hsdR*514 *rph*-1	[59]

For pUC57_noBsaI_xylBco, pUC57_AcBAD and pUC57_BaBAD, the codon-optimised DNA sequences provided by Biomatik can be found in Supplementary Data S1.

### Gene design and cloning

The genes encoding XylB from *P. putida* MT53 (UniProt: D5MPF3), BAD from *A. calcoaceticus* NCIB 8250 (AcBAD, UniProt: Q59096), and BAD from *B. ambifaria* MC40-6 (BaBAD, UniProt: B1Z4S6) were ordered as codon-optimised constructs from Biomatik (Supplementary Data S1). All three genes were cloned into the pBADcLIC vector using a recombination-based protocol. pBADcLIC was linearised via PCR using the primers pBADcLIC_Fd (GAAAATTTATACTTCCAAGGTCATCATCAC) and pBADcLIC_Rev (AGCAAATCCACCACCCAT). pUC57_noBsaI_XylBco, pUC57_AcBAD and pUC57_BaBAD were digested with BsaI, producing linear sequences with matching overhangs to the pBADcLIC vector. The linearised vector and inserts were ligated using ClonExpress II One Step Cloning Kit (Vazyme) according to the manufacturer's instructions, producing the pBADcLIC_XylBco, pBADcLIC_AcBAD and pBADcLIC_BaBAD vectors. The final expression constructs therefore contained an additional five amino acids (GGGFA) after the start codon and seventeen at the C-terminus (ENLYFQGHHHHHHHHHH) from the pBADcLIC vector. Subsequently, inverse PCR using the primers XylBco_repair_fd (GAAATAAAGGCAGCTATTGTACGGCAAAAAAATGG) and XylBco_repair_rev (AGCAAATCCACCACCCATGG) was used to remove the first six nucleotides of the XylB sequence, as we predicted that the start codon had been miscalled in the annotation, producing the pBADcLIC_XylBfix vector.

### Protein expression

For protein purification, a 20 ml overnight culture in LB was used to inoculate 1 L LB in a 2 L baffled flask, which was incubated at 37°C with shaking at 120 rpm until the culture reached an OD_600_ of ∼0.6. The culture was then induced by addition of arabinose to 0.01% (w/v). After 3 h, the culture was harvested by centrifugation at 5000***g*** for 20 min.

Protein purification was carried out in potassium phosphate (KPi) buffers at pH 7.2. Centrifuged cell pellets were resuspended in 30 ml KPi wash buffer (50 mM potassium phosphate pH 7.2, 200 mM sodium chloride, 20% (v/v) glycerol, 40 mM imidazole) and lysed by sonication (3 s on and 7 s off for 10 min, total sonication time 3 min). Lysates were then centrifuged at 27 000***g*** for 30 min and the lysates transferred into a fresh 50 ml Falcon tube. Proteins were purified via HPLC using a HisTrap HP 5 ml column (Cytiva); after loading, the column was washed with 10 column volumes of KPi wash buffer, followed by elution with KPi elution buffer (50 mM potassium phosphate pH 7.2, 200 mM sodium chloride, 20% (v/v) glycerol, 500 mM imidazole).

The IMAC purified proteins were buffer exchanged into the assay buffer (50 mM potassium phosphate pH 7.2, 200 mM sodium chloride) using a HiTrap Desalting column (GE healthcare). Protein concentrations were calculated by absorbance measured at 280 nm according to the respective extinction coefficients as determined using Expasy ProtParam [52].

### Enzyme activity assay

Reaction mixtures for the enzyme activity assay were prepared in assay buffer (50 mM potassium phosphate pH 7.2, 200 mM sodium chloride) with 0.2 mM of either NADH or NADPH and a range of concentrations of either furfural, hydroxymethylfurfural (HMF), benzaldehyde or hexanal. Purified enzymes in assay buffer were added to a final concentration of 0.25 μM. The reaction mixtures were incubated in a Nunc 2.0 microtitre plate with a plate seal at 37°C, shaking at 200 rpm in an Epoch 2 microplate spectrophotometer. The reaction was allowed to progress for an hour, measuring the reduction in absorbance at 340 nm every minute which corresponds to the oxidation of NADH and NADPH to NAD^+^ and NADP^+^, respectively. Utilisation of cofactor was calculated using the formula

ε=cv,

using an extinction coefficient of 6220 M^−1^ cm^−1^ and estimating a path length of 0.625 cm.

The *k*_cat_ and *K*_M_ values were calculated using GraphPad Prism v5. The fastest rate of cofactor utilisation was determined by plotting the converted NADH against time for each ligand at a specific concentration. The highest rates of conversion of NADH (V) from the first time point for each ligand concentration ([S]) were then plotted against the latter. A curve was fitted on each plot using non-linear regression for the estimation of V_max_. This analysis has been provided as ‘Supplementary Data S4.xlsx’. The following equations were used to determine the *K*_M_ and *k*_cat_:

$$V=V_{\max}*[S]/(K_{m}+[S])$$

$$V_{\max}=\mathrm{Et}*k_{\mathrm{cat}},$$

where Et = concentration of enzyme catalytic sites.

### Structural analysis

The structures for AcBAD and BaBAD were obtained from the Protein Data Bank [53] (1F8F [39] and 5TNX [40] respectively). The model of XylB was prepared on SWISS-MODEL (swissmodel.expasy.org [46]) using the known structure of AcBAD as the template for homology modelling. The QMEANDisCo global score [54] was calculated to be 0.82 ± 0.05 which suggests consistency with other models of a similar size. The derived GMQE (global model quality estimation) for the model [46] was determined to be 0.85, indicating high reliability. Structures were visualised using CCP4mg [55]. The SWISS-MODEL predicted structure of XylB is provided as ‘Supplementary Data S3.pdb’.

### Plate reader-based growth assay

Growth assays were carried out in M9 medium [56] with 2% (w/v) xylose, 2 mM MgSO_4_, and 100 µM CaCl_2_. M9 medium was modified by addition of 1 ml L^−1^ 1000× trace metals solution [57] (50 mM FeCl_3_, 20 mM CaCl_2_, 10 mM MnCl_2_, 10 mM ZnSO_4_, 2 mM CoCl_2_, 2 mM CuCl_2_, 2 mM NiCl_2_, 2 mM Na_2_MoO_4_, 2 mM Na_2_SeO_3_, 2 mM H_3_BO_3_, 0.1 M HCl). Additionally, citric acid was added to 0.05% (w/v) to prevent precipitation of iron, and casamino acids to 0.01% (w/v) in order to alleviate stress caused by recombinant protein expression [58]. This took the final pH of the medium to ∼4.6–4.7. Ampicillin was added to 100 µg/ml, and arabinose added to 0.01% (w/v) to induce expression of the target protein.

For the growth assays, strains were pre-cultured overnight in 5 ml modified M9-xylose medium. To ensure that the strains were at the same phase of growth at the beginning the experiment, the overnight cultures were used to inoculate over-day cultures of the same medium to an OD_600_ of 0.05. At an OD_600_ of ∼0.5, these cultures were diluted to an OD_600_ of 0.2 in the same medium. 96-well plates were prepared with 200 µl modified M9-xylose medium with furfural at the required concentration; wells were inoculated with 10 µl of the diluted cultures. The inoculated plates were incubated at 37°C in a Biotek Epoch 2 microplate spectrophotometer with double orbital shaking at 282 cpm for 48 h with OD_600_ being measured every 30 min. Data was plotted using OriginPro 2021b software.

### Supernatant analysis

For the supernatant analysis, cultures were grown in 250 ml flasks containing 30 ml modified M9-xylose medium with 10 mM furfural. Overnight cultures were prepared in 5 ml modified M9-xylose medium and used to inoculate the flasks to an OD_600_ of 0.05. Flasks were then grown at 37°C with shaking at 180 rpm; at 0, 2, 4, 6, 8, 10, and 12 h, 1 ml of the culture was harvested for OD_600_ measurement and analysis. Samples were centrifuged at 15 000***g*** for 1 min and the supernatants transferred to a fresh 1.5 ml centrifuge tube. Samples were stored at −20°C until required.

Supernatant samples were diluted 20-fold in deionised water and analysed for furfural, furfuryl alcohol and furoic acid. Standards for these compounds were prepared to 500 µM in deionised water. Additionally, a calibration curve was prepared with a mixture of all three compounds diluted to 500 µM, 300 µM, 100 µM, 50 µM, 25 µM and 10 µM. Samples were analysed on an Acquity I-Class UPLC equipped with a TUV detector (Waters, Wilmslow, U.K.). The column was an Acquity UPLC BEH C18, 130 Å, 1.7 µm, 2.1 mm × 100 mm (Waters). The mobile phase A) was 5% (v/v) methanol (Rathburn, Walkerburn, U.K.) with 0.1% (v/v) acetic acid (Fisher Optima), B) methanol with 0.1% (v/v) acetic acid. The elution gradient was 16% (v/v) B) isocratic for 2.5 min, which was ramped up to 100% B) over 0.3 min, left there for 0.1 min, and returned to 16% (v/v) B) over 0.4 min; total run time was 4 min. Flow rate was 0.5 ml/min, injection volume 2 µl, column temperature 45°C, and sample temperature 10°C. The UV detection wavelength was 230 nm. Standard curves of furfural, furoic acid and furfuryl alcohol (Sigma/Merck, Gillingham, U.K.) were constructed in water. Data analysis was undertaken with Xcalibur 4.0 (Thermo Scientific, Loughborough, U.K.). OriginPro 2021b software was used to plot the concentration and OD_600_ data and to estimate the rate of furfural conversion.

## Data Availability

The authors confirm that all relevant data has been provided either in the article or as Supplementary Material.

## Abbreviations

BAD: benzyl alcohol dehydrogenase
HMF: hydroxymethyl furfural
MIC: minimal inhibitory concentration
