# Supplementary material for: Improved furfural tolerance in Escherichia coli mediated by heterologous NADH-dependent benzyl alcohol dehydrogenases
Source: Biochem J. 2022 May 23;479(10):1045–58. doi: 10.1042/BCJ20210811 (PMC9162472; doi:10.1042/BCJ20210811)
Supplement: Supplementary Material 1 [file BCJ-479-1045-s1.pdf]

Improved furfural tolerance in *E. coli* mediated by heterologous NADH-dependent benzyl alcohol dehydrogenases

Benjamin James Willson[1], Reyme Herman[1], Swen Langer[2], Gavin Hugh Thomas[1]*

[1]Department of Biology and [2]Technology Facility, Department of Biology, University of York, York, UK, YO10 5DD

*Corresponding author

Supplementary Figure 1, Supplementary Data 1, 2 and 3, and Supplementary References

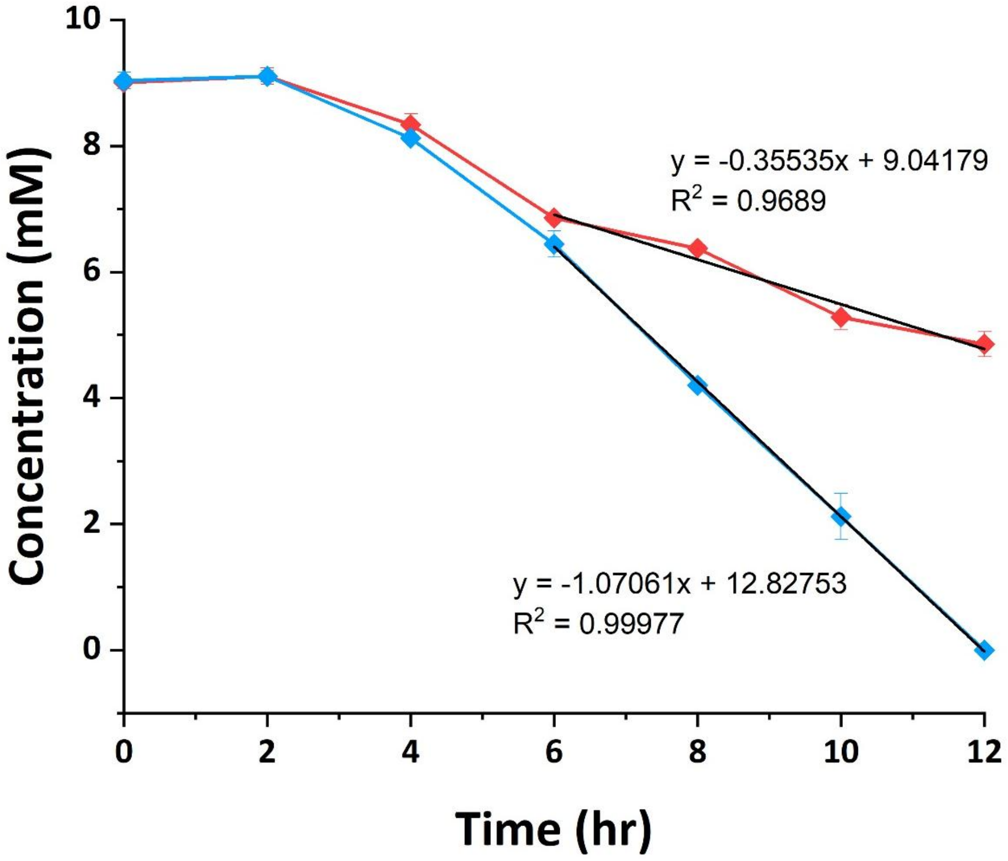

Supplementary Figure 1

Estimated furfural conversion rates for *E. coli* BW25113 pBADcLIC (red) or pBADcLIC_XylBfix (blue) as calculated from the 6 hr to 12 hr time points. Data is taken from Figure 6 and shows the concentration of furfural in the supernatant. Error bars show standard deviation with three biological replicates, except for the pBADclic 4 h and 6 h datapoints where N=2. OriginPro 2021b software was used to generate lines of best fit.

Supplementary Data

Supplementary Data 1: Codon-optimised sequences obtained from Biomatik. Blue sequences: Introduced sequences for BsaI cleavage and subsequent cloning into pBADcLIC. Yellow sequence subsequently removed from XylB based on our prediction of the correct start codon. Target sequences for inverse PCR using the XylBco_repair primers are underlined.

>XylB

GGTCTCTAACCATGGGTGGTGGATTTGCTGAAATGGAAATAAAGGCAGCTATTGTACGGCAAAAAAATGGCCCAT
TTTTACTTGAGCACGTGGCGTTGAATGAACCCGCAGAAGACCAGGTTCTGGTTCGCTTAGTTGCTACTGGCTTGT
GCCACACCGATCTTGTCTGCAGAGATCAGCATTACCCAGTCCCATTGCCCATGGTCTTCGGGCACGAGGGGGCCG
GAGTTGTTGAACGGGTCGGATCTGCTGTTAAGAAGGTCCAACCTGGCGATCACGTGGTACTGACATTTTACACAT
GTGGGTCGTGCGATGCATGTTTATCCGGGGACCCTACGTCGTGCGCGAACTCCTTTGGGCCTAATTTTATGGGTC
GGTCCGTGACGGGTGAATGCACAATACATGACCACCAGGGAGCAGAAGTTGGAGCTTCGTTCTTTGGGCAGAGCT
CATTCGCAACTTACGCGCTTTCCTACGAGCGCAATACAGTCAAGGTAACCAAAGATGTTCCGCTGGAATTACTTG
GCCCATTAGGCTGTGGTATACAAACGGGGGCGGGAAGTGTCTTGAACGCTTTGAATCCCCCGGCGGGTTCTAGCA
TAGCCATCTTTGGTGCAGGAGCTGTTGGATTGTCTGCCGTGATGGCGGCAGTCGTAGCAGGCTGCACAAAGATTA
TTGTGGTAGATGTGAAGGAGAATCGCTTAAAGCTGGCGGACGAGTTGGGGGCGACCCACGTCATCAATGCGGCCA
GCTCGGATCCGGTAGAAAAGATTAAGGAAATATGCGCCGGAGGGGTGCCGTACGTACTTGAAACTAGCGGTCTGC
CTAGCGTGCTTCAACAGGCAATTCTGTCATCCGCAATTGGAGGCGAAATTGGGATCGTAGGTGCCCCCCCGATGG
GAGCGACAATACCGGTGGACATTAACTTTCTGTTATTTAATCGGAAGTTACGTGGTATTGTGGAGGGCCAATCCA
TTTCCGATATTTTCATACCGCGCCTGGTAGAGCTTTATCGTCAAGGGAAATTTCCGTTTGACAAGCTTTTGAAAT
TCTATTCGTTTGATGAGATTAACCAGGCTGCTGAAGACAGCGAAAACGGTATTACATTGAAGCCGGTTCTGCGTA
TTTCCGAAAATTTATACTTCCAAGGTCAGAGACC

>Ac_BAD

GGTCTCTAACCATGGGTGGTGGATTTGCTAGCGAACTGAAAGATATTATCGCAGCAGTGACCCCGTGTAAAGGTG
CAGATTTTGAACTGCAGGCACTGAAAATTCGCCAGCCGCAGGGTGACGAAGTGCTGGTGAAAGTGGTTGCAACCG
GTATGTGCCATACCGATCTGATTGTGCGTGATCAGTATTATCCGGTGCCGCTGCCGGCAGTGCTGGGTCATGAAG
GCAGCGGTATTATTGAAGCCATTGGTCCGAATGTTACCGAACTGCAGGTGGGTGACCATGTTGTGCTGAGTTATG
GTTATTGTGGTAAATGCACCCAGTGTAATACCGGCAATCCGGCATATTGCAGTGAATTTTTCGGTCGCAATTTTA
GTGGCGCCGATAGTGAAGGTAATCATGCACTGTGCACCCATGATCAGGGTGTTGTTAATGATCATTTCTTTGCAC
AGAGCAGTTTTGCCACCTATGCCCTGAGCCGTGAAAATAATACCGTTAAAGTGACCAAAGATGTGCCGATTGAAC
TGCTGGGTCCGCTGGGCTGCGGTATTCAGACCGGCGCCGGTGCATGCATTAATGCCCTGAAAGTGACCCCGGCAA
GCAGTCTGGTGACCTGGGGCGCAGGTGCCGTTGGCCTGAGTGCACTGCTGGCCGCAAAAGTTTGCGGTGCCAGCA
TTATTATTGCCGTTGATATTGTGGAAAGCCGCCTGGAACTGGCAAAACAGCTGGGTGCCACCCATGTGATTAATA
GCAAAACCCAGGATCCGGTGGCAGCAATTAAGGAAATTACCGATGGCGGCGTGAATTTTGCCCTGGAAAGCACCG
GTCGTCCGGAAATTCTGAAACAGGGTGTGGATGCACTGGGCATTCTGGGCAAAATTGCCGTGGTGGGTGCACCGC
AGCTGGGTACCACCGCACAGTTTGATGTGAATGATCTGCTGCTGGGTGGCAAAACCATTCTGGGTGTGGTGGAAG
GTAGTGGCAGCCCGAAAAAATTCATTCCGGAACTGGTTCGTCTGTATCAGCAGGGCAAATTTCCGTTTGATCAGC
TGGTGAAATTTTATGCCTTTGATGAAATTAACCAGGCCGCAATTGATAGCCATAAAGGCATTACCCTGAAACCGA
TTATTAAGATTGCAGAAAATTTATACTTCCAAGGTCAGAGACC

>Ba_BAD

GGTCTCTAACCATGGGTGGTGGATTTGCTTACACCGAAAATGATACCCGTGCCGTTACCGCCGCAGTGGCACGTG
CCGCTGGTGCACCTTTTAGTATTGAACCGGCCCGCATTCGTGCCCCGCGTGGTGACGAAGTGCTGGTGCGTGTGG
TTGCAACCGGTCTGTGTCATACCGATCTGATTGTTCGCGATCAGTATTATCCGGTTCCGCTGCCGGCAGTGCTGG
GCCATGAAGGCGCAGGTGTGGTTGAAGCCGTTGGTCCGAATGTTAAAACCCTGGCAGCCGGCGATCATGTGGTTC
TGACCTATGGTGCCTGCGGCCATTGTGCCAGCTGTGCAGGCGGCCATGGTGCCTATTGTCGCCAGTTTTTCGCCC
TGAATTTTGGTGGTGCAGATGCCGATGGCCAGACCGCCCTGCGTGATGCCGCAGGTGAACCGCTGCATGATCATT

```
TCTTTGCACAGAGTAGCTTTGCAAGTTATGCCCTGGCCCGCGAAAATAATGCAATTAAGGTTCCGAAAGAAGCAC
CGCTGGAACTGCTGGGTCCGCTGGGTTGCGGTATTCAGACCGGTGCCGGCGCCGTGATTAATAGCCTGGCAGTTC
GCACCGGTAGCAGTTTTGCAAGTTTTGGCGCAGGTGCCGTTGGTATGAGTGCAGTGATGGCCGCCCGTATTGCAG
GCGCAACCACCATTATTGCCGTGGATATTGTGCCGAGCCGTCTGGCACTGGCACTGGAACTGGGTGCAACCCATG
CAATTAATAGCAAAGAAGTGGATGTGGTTGATGCCATTCGTGAAATTACCGGCGGCGGCGTGGATTATGCACTGG
AAAGTACCGGTCTGCCGGCAGTTCTGAGTCAGGGTATTGATGCCCTGGGCAGCCGTGGTACCATGGGCGTTGTTG
GTGCCCCGAAACTGGGTACCAAAGCAGAATTTGATGTTAATAGCCTGCTGCTGGGCGGTCATACCATTCGTGGTA
TTGTGGAAGGTGACAGTGTTCCGCAGACCTTTATTCCGCAGCTGGTTCAGCTGCATCTGCAGGGTCGTTTTCCGT
TTGATCGCCTGGTTAAATTTTATCCGCTGGAACAGATTAATCAGGCAGCCGCCGATAGTAGCAGTGGTATTACCC
TGAAACCGATTCTGCGCCTGCCGCATGAAAATTTATACTTCCAAGGTCAGAGACC
```

Supplementary Data 2: Alignment of XylB, AcBAD and BaBAD protein sequences using Clustal Omega [1]. Blue highlight represents the residues adjacent to the cofactor binding site discussed in Figure S1.

```
XylB       -------MEIKAAIVRQKNGPFLLEHVALNEPAEDQVLVRLVATGLCHTDLVCRDQHYPV     53
AcBAD      ---MSELKDIIAAVTPCKGADFELQALKIRQPQGDEVLVKVVATGMCHTDLIVRDQYYPV     57
BaBAD      MYTENDTRAVTAAVARAAGAPFSIEPARIRAPRGDEVLVRVVATGLCHTDLIVRDQYYPV     60
                    : **:.   .. * ::   :. *  *:***::****:*****: ***:***

XylB       PLPMVFGHEGAGVVERVGSAVKKVQPGDHVVLTFYTCGSCDACLSGDPTSCANSFGPNFM     113
AcBAD      PLPAVLGHEGSGIIEAIGPNVTELQVGDHVVLSYGYCGKCTQCNTGNPAYCSEFFGRNFS     117
BaBAD      PLPAVLGHEGAGVVEAVGPNVKTLAAGDHVVLTYGACGHCASCAGGHGAYCRQFFALNFG     120
           *** *:****:*::* :*  *. :  ******::  ** *  *  *. : * : *. **

XylB       GRSVTGECTIHDHQGAEVGASFFGQSSFATYALSYERNTVKVTKDVPLELLGPLGCGIQT     173
AcBAD      GADSEGNHALCTHDQGVVNDHFFAQSSFATYALSRENNTVKVTKDVPIELLGPLGCGIQT     177
BaBAD      GADADGQTALRDAAGEPLHDHFFAQSSFASYALARENNAIKVPKEAPLELLGPLGCGIQT     180
           * .  *: ::       :   **.*****:***: *.*::** *:.*:************

XylB       GAGSVLNALNPPAGSSIAIFGAGAVGLSAVMAAVVAGCTKIIVVDVKENRLKLADELGAT     233
AcBAD      GAGACINALKVTPASSLVTWGAGAVGLSALLAAKVCGASIIIAVDIVESRLELAKQLGAT     237
BaBAD      GAGAVINSLAVRTGSSFASFGAGAVGMSAVMAARIAGATTIIAVDIVPSRLALALELGAT     240
           ***: :*:*    .**:. :******:**::** :.*.: **.**:  .** ** :****

XylB       HVINAASSDPVEKIKEICAGGVPYVLETSGLPSVLQQAILSSAIGGEIGIVGAPPMGATI     293
AcBAD      HVINSKTQDPVAAIKEITDGGVNFALESTGRPEILKQGVDALGILGKIAVVGAPQLGTTA     297
BaBAD      HAINSKEVDVVDAIREITGGGVDYALESTGLPAVLSQGIDALGSRGTMGVVGAPKLGTKA     300
           *.**:   * *  *:**  *** :.**::* * :*.*.: : .  * :.:**** :*:.

XylB       PVDINFLLF-NRKLRGIVEGQSISDIFIPRLVELYRQGKFPFDKLLKFYSFDEINQAAED     352
AcBAD      QFDVNDLLLGGKTILGVVEGSGSPKKFIPELVRLYQQGKFPFDQLVKFYAFDEINQAAID     357
BaBAD      EFDVNSLLLGGHTIRGIVEGDSVPQTFIPQLVQLHLQGRFPFDRLVKFYPLEQINQAAAD     360
            .*:* **: .:.: *:***..  . ***.**.*: **:****:*:*** :::*****  *

XylB       SENGITLKPVLRIS-        366
AcBAD      SHKGITLKPIIKIA-        371
BaBAD      SSSGITLKPILRLPH        375
           * .******::::
```

Supplementary Data 3: Predicted model of XylB prepared on SWISS-MODEL using the structure of AcBAD (PDB ID: 1F8F) as the template. The model is provided as a .pdb file entitled ‘Supplementary Data 3.pdb’.

Supplementary Data 4: Michaelis-Menten curves for the enzymatic assay data as calculated using GraphPad 5. The data is provided as an .xlsx file entitled ‘Supplementary Data 4.xlsx’.

Supplementary Data 5: Data from Figure 5, showing the 24 hour time point for BW25113 pBADcLIC (upper) and BW25113 pBADcLIC_XylB (lower). Data shows the mean of three biological replicates, except for the furfural and furfuryl alcohol concentrations in the pBADcLIC 4 and 6 hour samples, which are the mean of two biological replicates.

pBADcLIC:

Time (hr)	Furfural (mM)	STDEV	Furfuryl alcohol (mM)	STDEV	OD_{600}	STDEV
**0**	9.01	0.0974	N/D	N/D	0.0443	0.00289
**2**	9.11	0.134	0.0969	0.168	0.0607	0.00252
**4**	8.34	0.167	0.723	0.189	0.110	0.00889
**6**	6.86	0.0188	1.04	0.0606	0.187	0.00710
**8**	6.38	0.0600	1.58	0.0637	0.247	0.0114
**10**	5.28	0.195	2.03	0.0497	0.266	0.00404
**12**	4.85	0.199	2.78	0.302	0.273	0.00265
**24**	N/D	N/D	6.22	0.157	4.41	0.597

XylB:

Time (hr)	Furfural (mM)	STDEV	Furfuryl alcohol (mM)	STDEV	OD_{600}	STDEV
**0**	9.03	0.130	N/D	N/D	0.0387	0.00808
**2**	9.10	0.0875	0.314	0.0239	0.0723	0.0160
**4**	8.12	0.0305	0.919	0.0361	0.104	0.00529
**6**	6.44	0.210	1.63	0.200	0.183	0.0146
**8**	4.20	0.0596	3.25	0.437	0.251	0.0238
**10**	2.12	0.370	5.42	0.202	0.270	0.00710
**12**	N/D	N/D	6.75	0.552	0.293	0.0178
**24**	N/D	N/D	6.54	0.382	5.25	0.172
